# Radial artery characteristics before and after intra-arterial administration of nitroglycerin and verapamil: an optical coherence tomography study

**DOI:** 10.3389/fmed.2026.1896387

**Published:** 2026-09-29

**Authors:** Senhu Wang, Jia Zhou, Saiying He, Hao Liu, Haotian Wang, Zixuan Li, Jincheng Guo

**Affiliations:** 1 Department of Emergency Medicine, Beijing Luhe Hospital, Capital Medical University, Beijing, China; 2 Department of Cardiology, Beijing Luhe Hospital, Capital Medical University, Beijing, China

**Keywords:** nitroglycerin, optical coherence tomography, radial artery, vasodilation, verapamil

## Abstract

**Background:**

Radial artery dimensions before and after intra-arterial vasodilator administration remain incompletely characterized along the vessel. This study used optical coherence tomography (OCT) to assess paired changes in mean lumen diameter.

**Methods:**

Twelve patients underwent baseline OCT 5 min after limited sheath insertion and post-administration OCT after intra-arterial nitroglycerin and verapamil, before coronary angiography. The full OCT-imaged radial artery span was divided into equal proximal, middle, and distal thirds before selecting anatomically matched analyzable portions. Analysis included 1,482 matched cross-sectional pairs (2,964 frames), with a cumulative matched analyzable length of 1,404.4 mm. Measurements at matched 1-mm intervals were aggregated within patients. Mean lumen diameter was the representative lumen metric; area and volume were supportive measurements.

**Results:**

Mean lumen diameter increased from 3.06 ± 0.49 to 3.35 ± 0.56 mm (mean paired change, 0.29 mm; 95% CI, 0.20–0.39; *P* < 0.001). The mean patient-level relative increase was 9.6%. Mean increases were observed in all three thirds in exploratory analyses.

**Conclusions:**

Mean radial artery lumen diameter was greater after intra-arterial nitroglycerin and verapamil than at baseline.

**Clinical trial registration:**

https://www.chictr.org.cn/hvshowproject.html?id=270,922&v=1.1, Chinese Clinical Trial Registry (ChiCTR) ChiCTR2500097574.

## Introduction

Radial artery caliber is an important consideration in transradial coronary procedures. A smaller radial artery may make access more difficult, increase the risk of radial spasm, and worsen sheath-to-artery mismatch (1–4). For this reason, intra-arterial vasodilator cocktails, usually containing nitroglycerin, verapamil, or both, are commonly administered during transradial procedures to facilitate catheter manipulation and reduce spasm (1, 2). Previous studies have reported radial artery enlargement after vasodilator administration, with diameter increases of approximately 0.2 to 0.5 mm and relative changes of 7% to 23%, although the magnitude of change varies according to the imaging modality, measurement method, and timing of assessment (5–7).

Most available data have come from ultrasound, angiography, or intravascular ultrasound. These modalities have provided useful information, but they do not fully describe how the radial artery changes along its length or how these changes translate into lumen volume. Optical coherence tomography (OCT), with its high spatial resolution, enables detailed assessment of radial artery lumen and wall structures and has been used to evaluate access-related injury and longer-term vascular remodeling after transradial procedures (8, 9). OCT evaluation of acute radial artery vasodilatory responses remains limited, particularly regarding longitudinally matched cross-sectional analysis and volume-based assessment across the analyzable radial artery.

In this study, we performed OCT before and after intra-arterial nitroglycerin and verapamil to quantify paired changes in radial artery dimensions using a predefined imaging protocol. Mean lumen diameter was selected as the representative lumen metric across the matched analyzable vessel length and by segment; lumen area and volume were supportive measurements.

## Methods

### Study design and population

This was a prospective, single-center study designed to assess acute radial artery vasodilation using OCT before and after intra-arterial administration of nitroglycerin and verapamil. Between March 2018 and November 2022, patients undergoing clinically indicated coronary OCT and radial artery OCT during the same transradial procedure were screened. Patients were prospectively enrolled in the present vasodilation study if paired radial artery OCT imaging before and after vasodilator administration was planned and both examinations were to be completed before coronary angiography. Patients were excluded if they had severe valvular heart disease, an estimated glomerular filtration rate of <30 mL/min/1.73 m^2^, a history of contrast-media allergy, were pregnant, or were unable to provide written informed consent. Of the 1,320 patients screened, 13 were prospectively enrolled and underwent paired radial artery OCT imaging at baseline and after intra-arterial nitroglycerin and verapamil; both examinations were completed before coronary angiography. These acquisitions yielded 45 matched baseline and post-administration OCT pullback pairs for image-level quality assessment. One patient contributing three matched pullback pairs was excluded because adequate longitudinal anatomical matching could not be achieved. The final analysis included 12 patients and 42 matched pullback pairs; six patients contributed four pairs and six contributed three pairs. Patient selection and the disposition of matched OCT pullback pairs are summarized in Figure 1. The remaining 1,307 patients underwent radial artery OCT only after coronary angiography or intervention and were therefore not eligible for the present paired vasodilation study. The study was approved by the Ethics Committee of Beijing Luhe Hospital (No. 2018-LHKS-021) and was conducted in accordance with the Declaration of Helsinki. All enrolled participants provided written informed consent.

**Figure 1:**
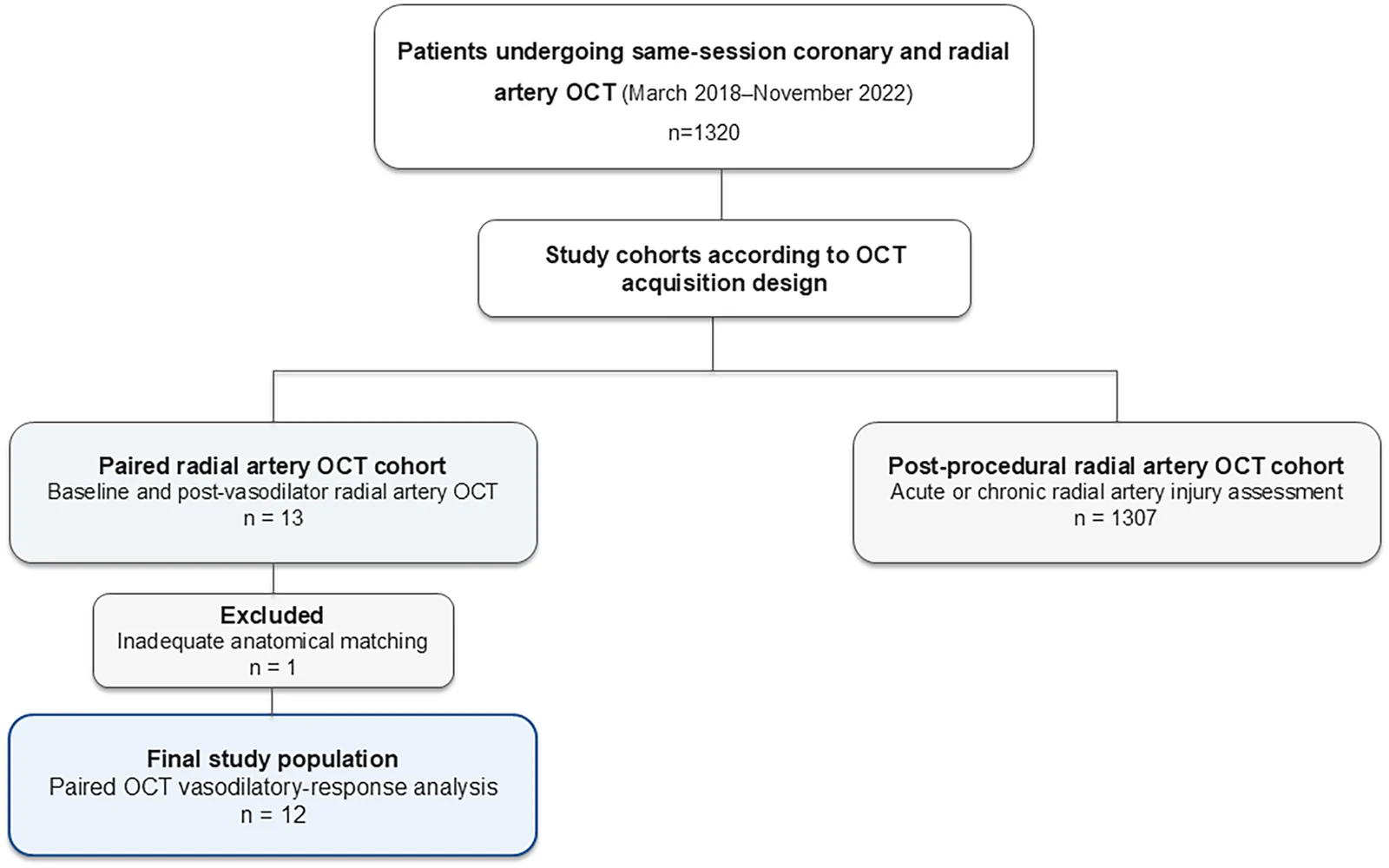
Study population and OCT pullback selection. Flow diagram showing patient screening and the disposition of matched baseline and post-administration radial artery OCT pullback pairs. OCT, optical coherence tomography.

Regular nitrate and calcium channel blocker use, including the specific agent, was obtained from the medical record. The timing of the last outpatient dose was not consistently available.

### Trial registration

This study was part of a broader investigator-initiated radial artery OCT research program registered with the Chinese Clinical Trial Registry under the title “Mapping the Distribution of Radial Artery Diameter, Atherosclerosis, and Acute and Chronic Injury Using Optical Coherence Tomography” (ChiCTR2500097574; February 15, 2025). Registration was retrospective because enrollment for the present study had ended in November 2022. Prospective registration was not completed because the investigators initially regarded the study as exploratory imaging research and did not recognize that its interventional component required prospective registration.

### OCT imaging protocol

After successful right radial artery puncture, a 6-Fr, 16-cm sheath (Terumo, Tokyo, Japan) was advanced approximately 2 cm into the artery to maintain vascular access while minimizing direct mechanical interaction between the sheath and the radial artery segment evaluated by OCT. A similar limited sheath-insertion approach has been described previously (10). Unfractionated heparin 3,000 U was then administered through the sheath. To minimize potential contrast-induced changes in baseline radial vasomotor tone, radial angiography was not performed before baseline OCT imaging.

For fluoroscopic landmark referencing, a radiopaque ruler was positioned along the forearm, with the radial artery origin near the level of the radial head serving as the proximal anatomical reference. A towel clamp was placed at the skin entry site as an external landmark, and the sheath tip, located approximately 2 cm proximal to the skin entry site, served as the distal procedural landmark (Figure 2). A 0.014-inch coronary guidewire was advanced into the brachial artery to facilitate OCT catheter placement. After the OCT catheter had been positioned, the guidewire was completely withdrawn, and imaging was performed off-wire.

**Figure 2:**
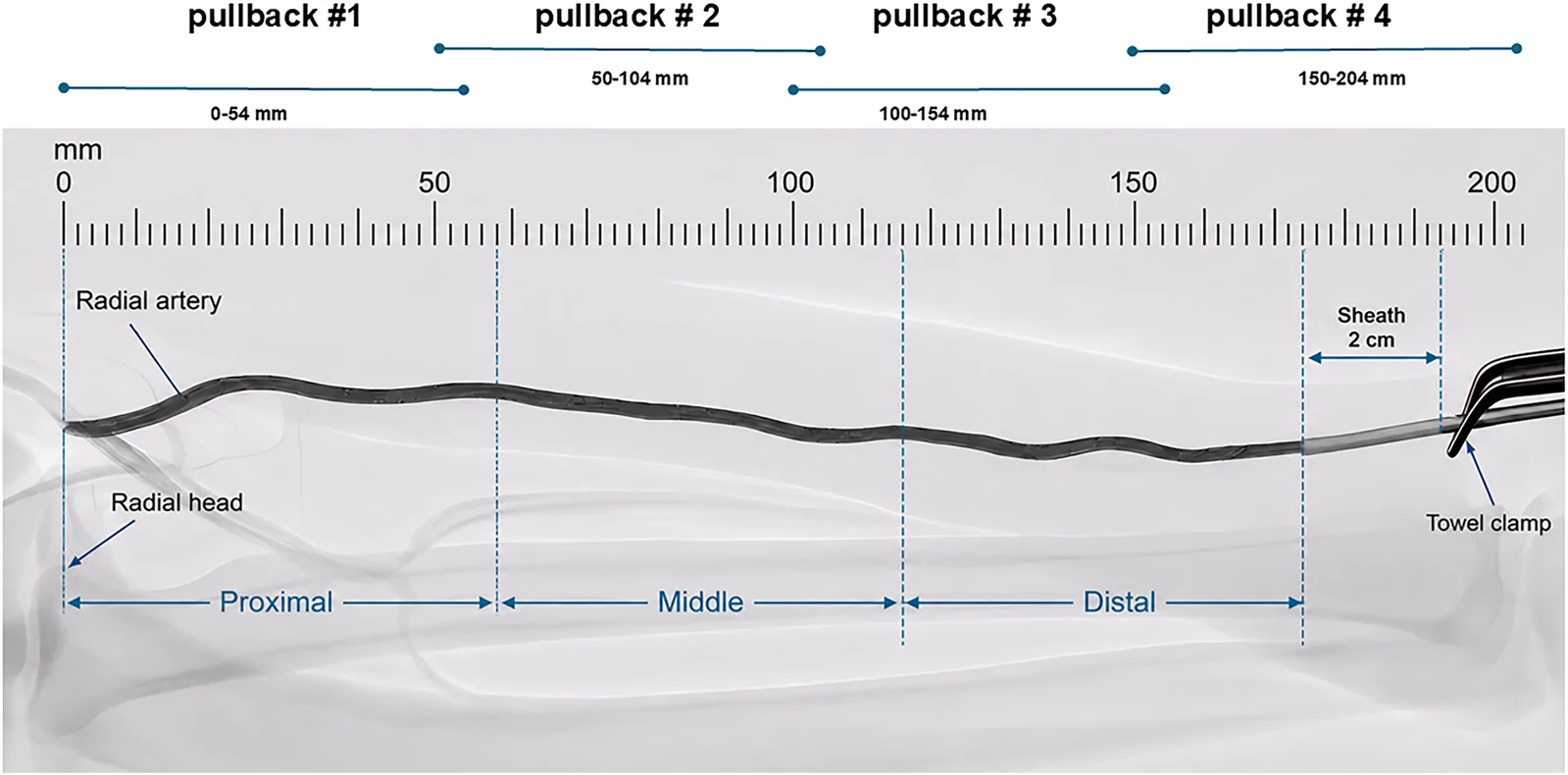
Radial artery OCT acquisition and longitudinal segmentation. Three or four sequential 54-mm OCT pullbacks were acquired from the radial artery origin toward the sheath tip, depending on arterial length. Adjacent pullbacks overlapped by approximately 4 mm and were manually aligned using shared OCT landmarks, with the fluoroscopic ruler providing an external localization reference. The schematic illustrates a 174-mm radial artery span requiring four pullbacks. This span was divided into equal proximal, middle, and distal thirds of 58 mm each, with boundaries at 58 and 116 mm. The matched analyzable portions retained within each third are not depicted. The towel clamp marked the skin entry site; the sheath tip was approximately 20 mm proximal to it. OCT, optical coherence tomography.

Five minutes after partial sheath insertion, a baseline series of three or four sequential OCT pullbacks, depending on the individual radial artery length, was acquired during saline flushing to cover the predefined segment from the radial artery origin to the sheath tip. Adjacent pullbacks overlapped by approximately 4 mm. After intra-arterial administration of 200 μg nitroglycerin and 2.5 mg verapamil, a second OCT series was planned to begin approximately 2 min after administration. This protocol target was selected as an early assessment time point because a previous radial IVUS study using the same doses demonstrated significant lumen-volume enlargement within 1 min (5), while minimizing the potential for additional time-dependent changes in vascular tone and procedural delay. The radiopaque ruler and the same fluoroscopic landmarks were used to reproduce the baseline acquisition positions and anatomical coverage during the second acquisition. After completion of radial artery OCT imaging, the sheath was fully advanced, and clinically indicated coronary angiography and coronary OCT imaging were performed, followed by coronary intervention as required (Figure 3). Drug-completion time was not recorded separately, so the exact elapsed time from completion of administration could not be reconstructed.

**Figure 3:**
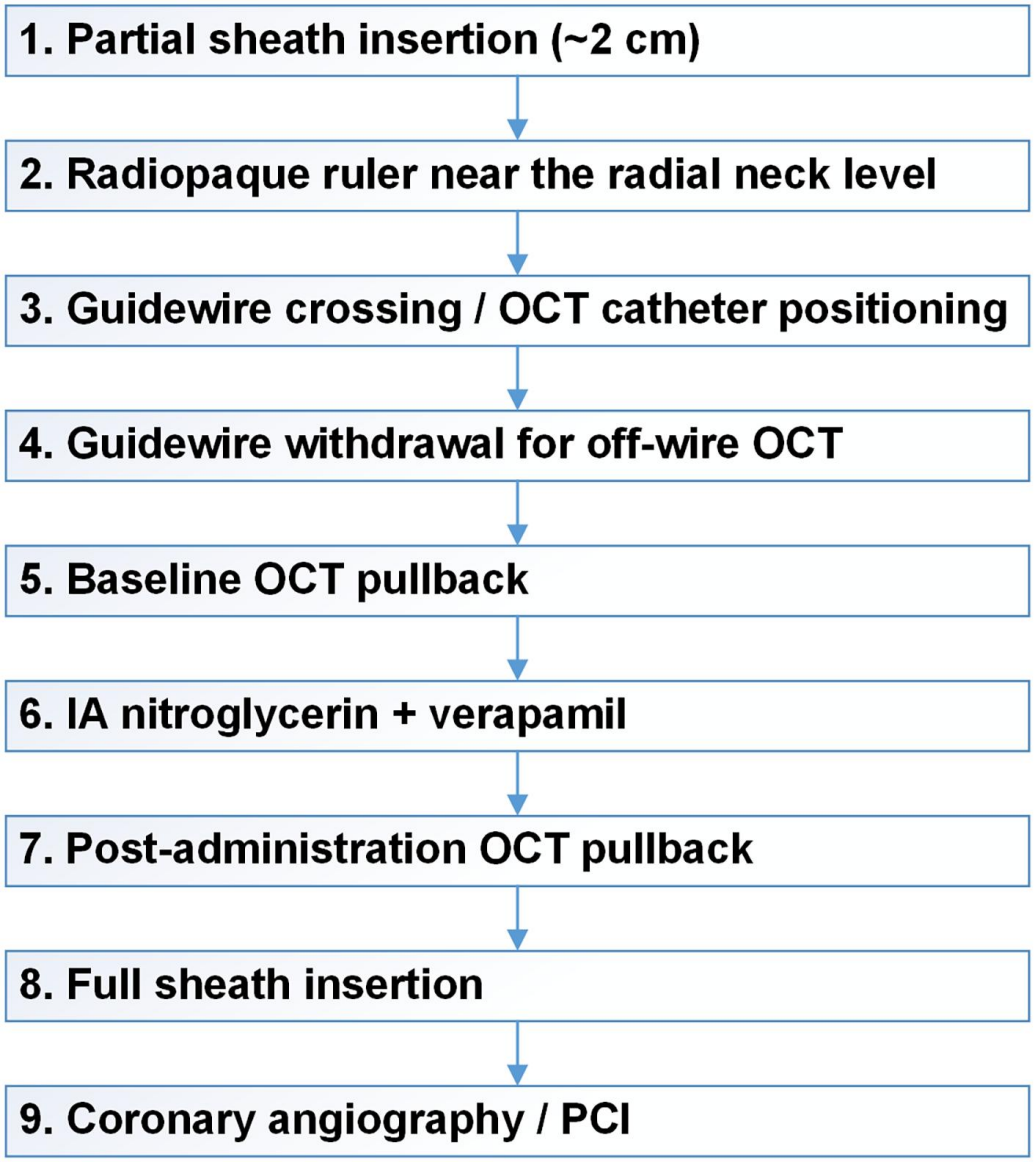
Procedural workflow for baseline and post-administration OCT pullbacks. A baseline series of OCT pullbacks was performed after partial sheath insertion and before coronary angiography or intervention. After intra-arterial nitroglycerin and verapamil administration, a second series of OCT pullbacks was obtained over the same predefined radial artery segment using identical fluoroscopic landmarks. The sheath was then fully advanced for coronary angiography or intervention.

All OCT pullbacks were performed with automatic pullback at 20 mm/s using the C7XR FD-OCT system (Abbott Vascular, Santa Clara, CA, USA), with a pullback length of 54 mm for each run.

Acquisition times were retrieved retrospectively from OCT workstation timestamps. Each complete OCT series began with the first pullback and ended with the final pullback. The inter-series interval extended from the end of the baseline series to the start of the post-administration series. The midpoint-to-midpoint interval was calculated as the difference between the midpoints of the two series.

### OCT image matching, segment definition, and quantitative analysis

Baseline and post-administration OCT pullbacks were reviewed offline. Pullbacks were included only when image quality was adequate and longitudinal anatomic matching between the two runs was sufficient for reliable quantitative comparison.

For each pullback, the analyzable length was defined as the imaged radial artery segment with adequate image quality for measurement. The baseline and post-administration pullbacks were matched using OCT-derived anatomical landmarks, including visible side branches, together with predefined fluoroscopic references (radiopaque ruler, external skin marker, and sheath-tip reference plane), to identify corresponding cross-sections along the same longitudinal vessel segment. The matched analyzable length was defined as the overlapping segment that was both analyzable and anatomically matched in the baseline and post-administration pullbacks.

For each patient, the full OCT-imaged radial artery span was divided into equal proximal, middle, and distal thirds according to longitudinal distance (Figure 2). Within each predefined third, matched analyzable portions were retained on the basis of adequate image quality and anatomical correspondence between baseline and post-administration pullbacks. Quantitative analysis was performed at 1-mm intervals along these portions. Marker-defined subsegments, such as M1 and M2, were used when present to maintain anatomical correspondence and were pooled within the corresponding third.

At each matched cross-section, OCT-derived measurements included mean, minimum, and maximum lumen diameter, lumen area, internal elastic lamina (IEL) area, and external elastic lamina (EEL) area. Mean lumen diameter was obtained directly from the Abbott OCT analysis software. Medial area was calculated as EEL area minus IEL area. For EEL-derived measurements, the EEL was considered identifiable when it was sufficiently visualized for reliable delineation of the outer vessel boundary. Only matched cross-sectional pairs with identifiable EEL in both baseline and post-administration acquisitions were included in paired EEL area and medial area analyses.

Lumen volume was calculated as the sum of lumen areas at 1-mm intervals along the matched analyzable length. Segmental lumen volume was defined as the sum of all matched marker-defined subsegments within the proximal, middle, or distal radial artery, and patient-level lumen volume was calculated as the sum of the three segmental volumes.

Mean lumen diameter was selected as the representative lumen metric. Lumen area and volume were derived from the same traced contour and treated as supportive measurements rather than independent outcomes.

As an exploratory measure of sheath-to-artery mismatch, the distal minimum lumen diameter-to-sheath outer diameter ratio was calculated by dividing the narrowest distal minimum lumen diameter by the outer diameter of the 6-Fr sheath (2.62 mm).

### Measurement reproducibility

Offline quantitative OCT analysis was performed by two experienced observers who were blinded to clinical data and to each other's measurements. For the reproducibility assessment, each observer independently analyzed 10 randomly selected cross-sectional frames per patient (120 frames in total). Equal sampling provided balanced patient representation; the sample size was pragmatic and was not based on a prespecified precision calculation. Intraclass correlation coefficients (ICCs) were reported with 95% confidence intervals. Observer-defined longitudinal matching was assessed separately in all 24 reconstructed datasets (12 patients, two imaging conditions). Both assessments used existing images and did not evaluate repeat acquisition. Agreement was evaluated using a two-way random-effects, absolute-agreement, single-measure model [ICC (1, 2)]. For longitudinal matching, proximal and distal matching points and matched segment length were recorded, and mean absolute between-observer differences in frame number were calculated. Discrepancies in measurements used for the final analysis were resolved by consensus.

### Statistical analysis

Statistical analyses were performed using R software version 4.5 (R Foundation for Statistical Computing, Vienna, Austria); patient-level recalculations and timing analyses used Python version 3.12.13 with SciPy version 1.17.0. Continuous variables are presented as mean ± standard deviation or median (interquartile range), as appropriate, and categorical variables as counts and percentages. Paired comparisons used the two-sided exact Wilcoxon signed-rank test. *P* values < 0.05 were considered statistically significant. No adjustment for multiple comparisons was performed; segmental findings were exploratory.

All statistical comparisons used the patient as the independent unit of analysis. Measurements from matched cross-sections were averaged within each patient for whole-vessel and segment-specific estimates, except lumen volume, which was summed at 1-mm intervals. Each patient contributed one paired estimate per measurement and, in segmental analyses, per third. Whole-vessel means reflected all retained frames within the patient, rather than an unweighted average of the three thirds. Two-sided 95% confidence intervals were calculated from patient-level estimates using the t distribution. Intervals for paired changes were based on within-patient differences.

Absolute change was calculated as the post-administration value minus the baseline value. Relative change was calculated as [(post-administration value−baseline value)/baseline value] × 100%. For segmental analyses, OCT-derived measurements within the proximal, middle, and distal thirds were calculated separately for each patient and compared between baseline and post-administration conditions, with each patient contributing one paired observation per segment.

The distal minimum lumen diameter-to-sheath outer diameter ratio was analyzed as an exploratory parameter of sheath-to-artery mismatch.

For each patient, mean lumen diameter was calculated across all matched analyzable frames at baseline and after administration. Absolute and relative changes were related to the midpoint-to-midpoint interval using Spearman correlation. The inter-series interval was examined as a supporting timing measure. These patient-level analyses were *post hoc* and exploratory.

## Results

As shown in Figure 1, 1,320 patients undergoing coronary and radial artery OCT during the same transradial procedure were screened. According to the predefined OCT acquisition design, 13 patients underwent paired radial artery OCT imaging before and after intra-arterial vasodilator administration. One patient was excluded because of inadequate longitudinal anatomical matching, yielding a final cohort of 12 patients for paired before-and-after analysis.

Baseline clinical characteristics and invasive hemodynamic parameters before and after vasodilator administration are summarized in Table 1. Systolic arterial pressure decreased from 125.3 ± 21.2 mmHg at baseline to 107.3 ± 18.7 mmHg after vasodilator administration. Background nitrate and calcium channel blocker use is summarized in Table 1.

**Table 1:** Baseline clinical characteristics and invasive hemodynamic parameters.

Variable	Overall (*n* = 12)
Clinical characteristics	
Age, years	42.7 ± 12.2
Male sex, *n* (%)	10 (83.3)
Hypertension, *n* (%)	6 (50.0)
Diabetes mellitus, *n* (%)	1 (8.3)
Hyperlipidemia, *n* (%)	8 (66.7)
Peripheral vascular disease, *n* (%)	0 (0)
History of renal insufficiency, *n* (%)	0 (0)
Current smoking, *n* (%)	8 (66.7)
Medication use	
Aspirin use, *n* (%)	9 (75.0)
Clopidogrel use, *n* (%)	8 (66.7)
Statin use, *n* (%)	8 (66.7)
Regular nitrate use, *n* (%)	8 (66.7)
Regular calcium channel blocker use, *n* (%)	2 (16.7)
ACEI/ARB use, *n* (%)	2 (16.7)
Hemodynamic parameters	
Baseline systolic arterial pressure, mmHg	125.3 ± 21.2
Baseline diastolic arterial pressure, mmHg	70.3 ± 10.5
Baseline heart rate, bpm	76.8 ± 12.4
Post-administration systolic arterial pressure, mmHg	107.3 ± 18.7
Post-administration diastolic arterial pressure, mmHg	66.7 ± 13.9
Post-administration heart rate, bpm	77.5 ± 15.1
Clinical presentation	
Unstable angina, *n* (%)	7 (58.3)
Stable angina, *n* (%)	5 (41.7)

Values are mean ± SD or *n* (%). Hemodynamic parameters were measured before and after intra-arterial vasodilator administration. ACEI, angiotensin-converting enzyme inhibitor; ARB, angiotensin receptor blocker; bpm, beats per minute. Calcium channel blockers were amlodipine and nifedipine (one patient each); no patient regularly used verapamil or diltiazem.

A total of 3,097 anatomically corresponding OCT cross-sectional frames were initially identified. After exclusion of 133 frames (4.3%) because of image-quality limitations, including motion artifact (*n* = 23) and inadequate blood clearance (*n* = 110), 2,964 matched OCT cross-sectional frames (1,482 baseline and 1,482 post-administration frames) were retained for quantitative analysis. The proximal, middle, and distal thirds contained 904, 1,128, and 932 retained frames, respectively. Across the 12 patients, the cumulative matched analyzable lengths retained within the predefined proximal, middle, and distal thirds were 422.8, 538.8, and 442.8 mm, respectively. The total was 1,404.4 mm, corresponding to a mean matched analyzable length of 117 mm per patient.

Among 1,482 anatomically matched cross-sectional pairs, comprising 2,964 individual OCT frames, the EEL was identifiable in 1,453 baseline frames (98.0%) and 1,449 post-administration frames (97.8%). The EEL was identifiable in both members of 1,431 matched pairs (96.6%), which were included in paired EEL area and medial area analyses.

Contour-tracing agreement and observer-defined longitudinal matching are summarized in Supplementary Tables 1–3. These analyses assess agreement within the available images, not variability between independently acquired OCT series.

The baseline and post-administration OCT series lasted 118.8 ± 48.9 and 94.7 ± 38.5 s, respectively. The inter-series interval was 142.8 ± 18.8 s (range, 119–171), and the midpoint-to-midpoint interval was 249.5 ± 40.2 s (range, 182.5–296.0).

No evidence of a correlation with absolute diameter change was detected for either the midpoint-to-midpoint interval (*ρ* = 0.084, *P* = 0.795) or the inter-series interval (*ρ* = −0.105, *P* = 0.746). Corresponding correlations with relative diameter change were *ρ* = 0.028 (*P* = 0.931) and *ρ* = −0.084 (*P* = 0.795).

An illustrative matched cross-sectional OCT example is shown in Figure 4. Mean lumen diameter increased from 3.06 ± 0.49 mm at baseline to 3.35 ± 0.56 mm after administration (mean paired change, 0.29 mm; 95% CI, 0.20–0.39; mean relative change, 9.6%; *P* < 0.001; Table 2 and Figure 5). Supportive lumen area and volume measurements are reported in Tables 2, 3. IEL, EEL, and medial area measurements are also reported in these tables; EEL and medial area estimates use the 1,431 pairs with assessable EEL at both time points.

**Figure 4:**
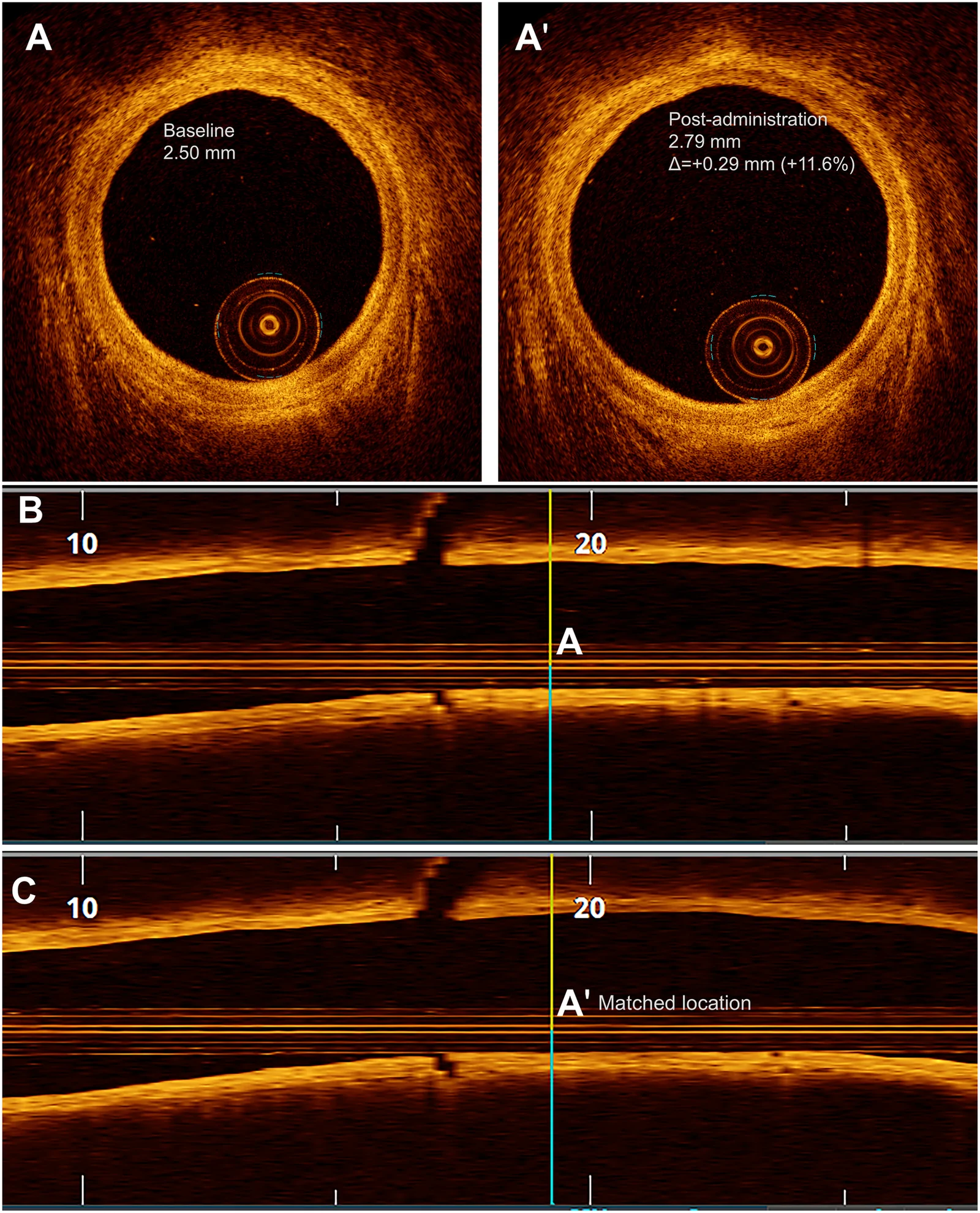
Paired radial artery OCT images at baseline and after intra-arterial nitroglycerin and verapamil. **(A)** and **(A′)** show cross-sectional images at the matched location at baseline and after administration, respectively. **(B)** and **(C)** show the corresponding longitudinal views; vertical markers indicate the locations of A and A′. At this location, mean lumen diameter increased from 2.50 to 2.79 mm (11.6%).

**Table 2:** Patient-level changes in radial artery OCT measurements before and after intra-arterial nitroglycerin and verapamil.

Variable	Baseline	Post-administration	Absolute change	95% CI for mean change	Relative change (%)	*P* value
Mean lumen diameter, mm	3.06 ± 0.49	3.35 ± 0.56	0.29 ± 0.15	0.20 to 0.39	9.59 ± 4.78	<0.001
Minimum lumen diameter, mm	2.97 ± 0.48	3.26 ± 0.54	0.28 ± 0.15	0.19 to 0.38	9.55 ± 4.82	<0.001
Maximum lumen diameter, mm	3.14 ± 0.51	3.44 ± 0.57	0.30 ± 0.16	0.20 to 0.40	9.61 ± 4.76	<0.001
Lumen area, mm^2^	7.61 ± 2.35	9.10 ± 2.86	1.49 ± 0.87	0.94 to 2.04	19.85 ± 10.01	<0.001
Lumen volume, mm^3^	934.98 ± 311.40	1,119.37 ± 373.61	184.39 ± 106.39	116.79 to 251.99	19.85 ± 10.01	<0.001
IEL area, mm^2^	8.04 ± 2.33	9.56 ± 2.86	1.52 ± 0.89	0.96 to 2.09	18.93 ± 9.42	<0.001
EEL area, mm^2^	9.72 ± 2.63	11.28 ± 3.21	1.56 ± 0.90	0.99 to 2.13	15.79 ± 7.63	<0.001
Medial area, mm^2^	1.69 ± 0.48	1.72 ± 0.51	0.03 ± 0.04	0.001 to 0.057	1.50 ± 2.33	0.034

Values are mean ± SD across 12 patients, except 95% CIs and *P* values. Absolute change = post-administration−baseline; relative change = [(post-administration−baseline)/baseline] × 100%, calculated for each patient before cohort summarization. Two-sided 95% CIs for mean changes use the t distribution; *P* values use the two-sided exact Wilcoxon signed-rank test and are unadjusted for multiple comparisons. Lumen and IEL measurements used 1,482 matched cross-sectional pairs. EEL and medial area analyses used the 1,431 pairs with assessable EEL at both time points; 51 pairs were excluded from these two analyses only. Medial area was calculated as EEL area minus IEL area within the same retained frames. Each patient contributed one paired estimate per measurement. Mean lumen diameter was obtained directly from the Abbott OCT analysis software. Minimum and maximum lumen diameters are averages of the corresponding cross-sectional measurements. Lumen volume was calculated as the sum of areas sampled at 1-mm intervals. Lumen area and volume are supportive measurements derived from the same contour. CI, confidence interval; EEL, external elastic lamina; IEL, internal elastic lamina; OCT, optical coherence tomography; SD, standard deviation.

**Table 3:** Segmental changes in radial artery OCT measurements before and after intra-arterial nitroglycerin and verapamil.

Variable	Segment	Baseline	Post-administration	Absolute change	95% CI for mean change	Relative change (%)	*P* value
Mean lumen diameter, mm	Proximal	3.37 ± 0.64	3.66 ± 0.59	0.28 ± 0.15	0.19 to 0.38	9.03 ± 5.54	<0.001
	Middle	3.02 ± 0.45	3.32 ± 0.56	0.30 ± 0.19	0.18 to 0.42	9.82 ± 5.56	<0.001
	Distal	2.80 ± 0.46	3.09 ± 0.57	0.29 ± 0.20	0.16 to 0.42	10.13 ± 6.71	<0.001
	Proximal	3.29 ± 0.62	3.56 ± 0.57	0.27 ± 0.14	0.18 to 0.36	8.85 ± 5.30	<0.001
Minimum lumen diameter, mm	Middle	2.94 ± 0.44	3.23 ± 0.54	0.30 ± 0.18	0.18 to 0.41	10.02 ± 5.81	<0.001
	Distal	2.72 ± 0.45	2.99 ± 0.55	0.27 ± 0.20	0.14 to 0.40	9.85 ± 6.97	<0.001
	Proximal	3.47 ± 0.67	3.76 ± 0.60	0.29 ± 0.16	0.19 to 0.40	9.18 ± 5.74	<0.001
Maximum lumen diameter, mm	Middle	3.10 ± 0.47	3.40 ± 0.57	0.30 ± 0.19	0.18 to 0.42	9.57 ± 5.50	<0.001
	Distal	2.88 ± 0.47	3.19 ± 0.58	0.31 ± 0.20	0.18 to 0.44	10.46 ± 6.53	<0.001
	Proximal	9.27 ± 3.36	10.76 ± 3.19	1.49 ± 0.80	0.98 to 2.00	18.91 ± 12.40	<0.001
Lumen area, mm^2^	Middle	7.32 ± 2.12	8.89 ± 2.93	1.57 ± 1.12	0.86 to 2.27	20.63 ± 11.92	<0.001
	Distal	6.35 ± 1.98	7.74 ± 2.68	1.40 ± 1.08	0.71 to 2.08	21.16 ± 14.59	<0.001
	Proximal	348.21 ± 146.71	405.83 ± 157.50	57.62 ± 41.46	31.28 to 83.96	18.91 ± 12.40	< 0.001
Lumen volume, mm^3^	Middle	345.10 ± 116.53	416.42 ± 145.27	71.31 ± 49.99	39.55 to 103.07	20.63 ± 11.92	<0.001
	Distal	241.66 ± 87.37	297.12 ± 120.26	55.46 ± 47.31	25.40 to 85.52	21.16 ± 14.59	< 0.001
	Proximal	9.69 ± 3.36	11.21 ± 3.24	1.52 ± 0.77	1.03 to 2.01	18.06 ± 11.09	<0.001
IEL area, mm^2^	Middle	7.71 ± 2.10	9.30 ± 2.91	1.59 ± 1.11	0.88 to 2.29	19.78 ± 11.23	<0.001
	Distal	6.83 ± 1.96	8.25 ± 2.68	1.42 ± 1.11	0.71 to 2.12	19.97 ± 14.29	< 0.001
	Proximal	11.69 ± 3.79	13.18 ± 3.68	1.50 ± 0.74	1.03 to 1.97	14.49 ± 8.95	<0.001
EEL area, mm^2^	Middle	9.33 ± 2.40	10.94 ± 3.27	1.61 ± 1.15	0.88 to 2.34	16.40 ± 9.36	<0.001
	Distal	8.34 ± 2.24	9.82 ± 3.07	1.49 ± 1.20	0.72 to 2.25	16.78 ± 12.07	< 0.001
	Proximal	2.00 ± 0.59	1.97 ± 0.56	−0.03 ± 0.09	−0.085 to 0.033	−1.05 ± 4.11	0.910
Medial area, mm^2^	Middle	1.63 ± 0.48	1.65 ± 0.48	0.02 ± 0.10	−0.041 to 0.085	1.45 ± 5.57	0.519
	Distal	1.50 ± 0.40	1.57 ± 0.49	0.07 ± 0.11	0.001 to 0.141	3.80 ± 5.84	0.021

Values are mean ± SD across 12 patients per segment, except 95% CIs and *P* values. Absolute and relative changes were calculated within patients; 95% CIs use the t distribution. *P* values use the two-sided exact Wilcoxon signed-rank test within each segment and are unadjusted for multiple comparisons. Lumen and IEL measurements used 452 proximal, 564 middle, and 466 distal matched pairs. EEL and medial area analyses used 441, 550, and 440 pairs, respectively (total, 1,431); both time points had assessable EEL. Medial area was calculated from EEL and IEL areas in the same retained frames. All analyses used patients, not frames, as the independent units. Segmental findings are exploratory. Minimum and maximum lumen diameters are averages of the corresponding cross-sectional measurements. Lumen area and volume are supportive measurements. CI, confidence interval; EEL, external elastic lamina; IEL, internal elastic lamina; OCT, optical coherence tomography; SD, standard deviation.

**Figure 5:**
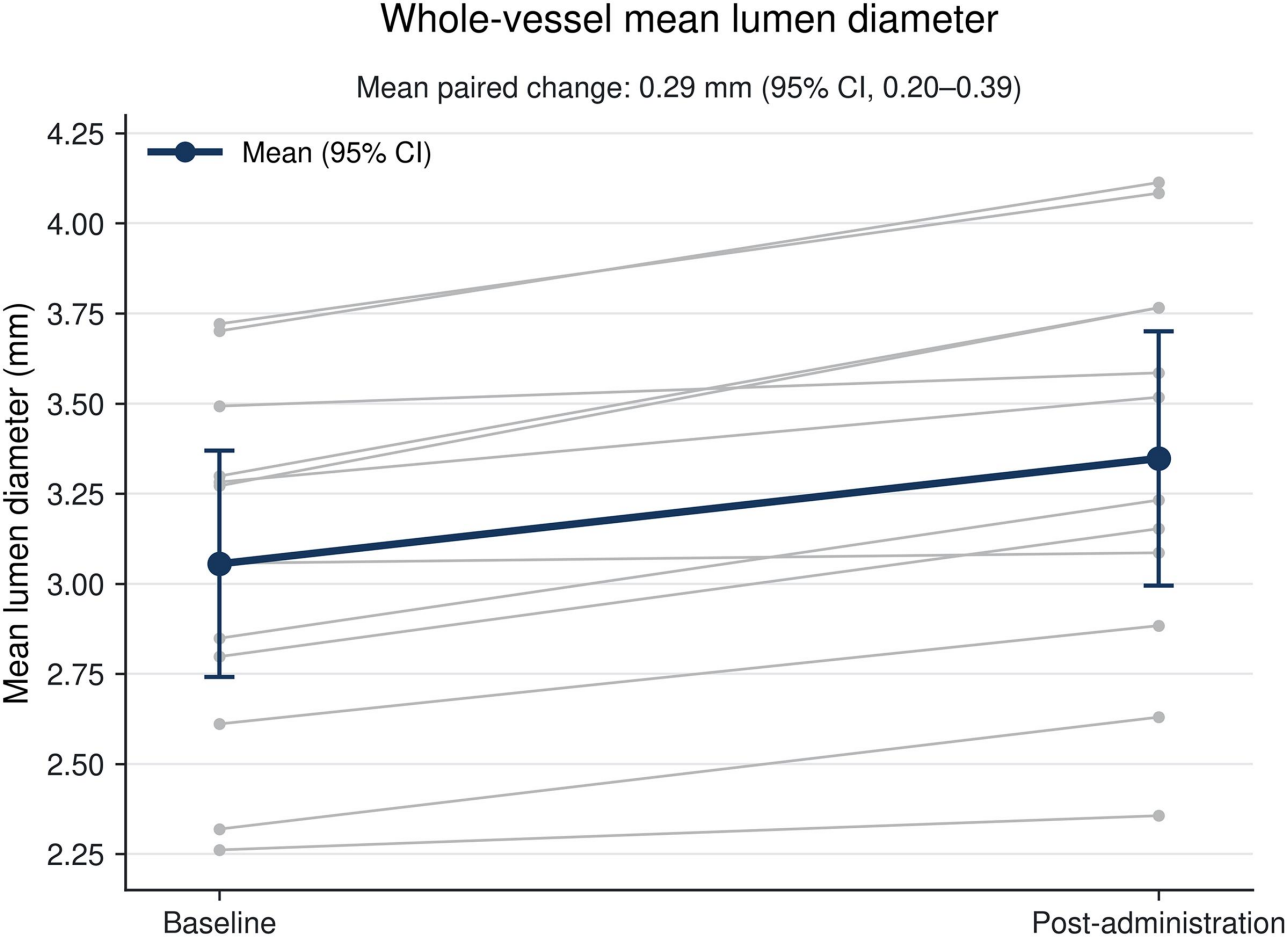
Whole-vessel change in mean radial artery lumen diameter before and after intra-arterial nitroglycerin and verapamil. Gray lines represent paired patient-level observations. Navy symbols and error bars show the mean and 95% confidence interval at each time point. The annotated interval refers to the mean within-patient change.

In exploratory segmental analyses, mean lumen diameter increased after administration in the proximal, middle, and distal thirds (Table 3 and Figure 6).

**Figure 6:**
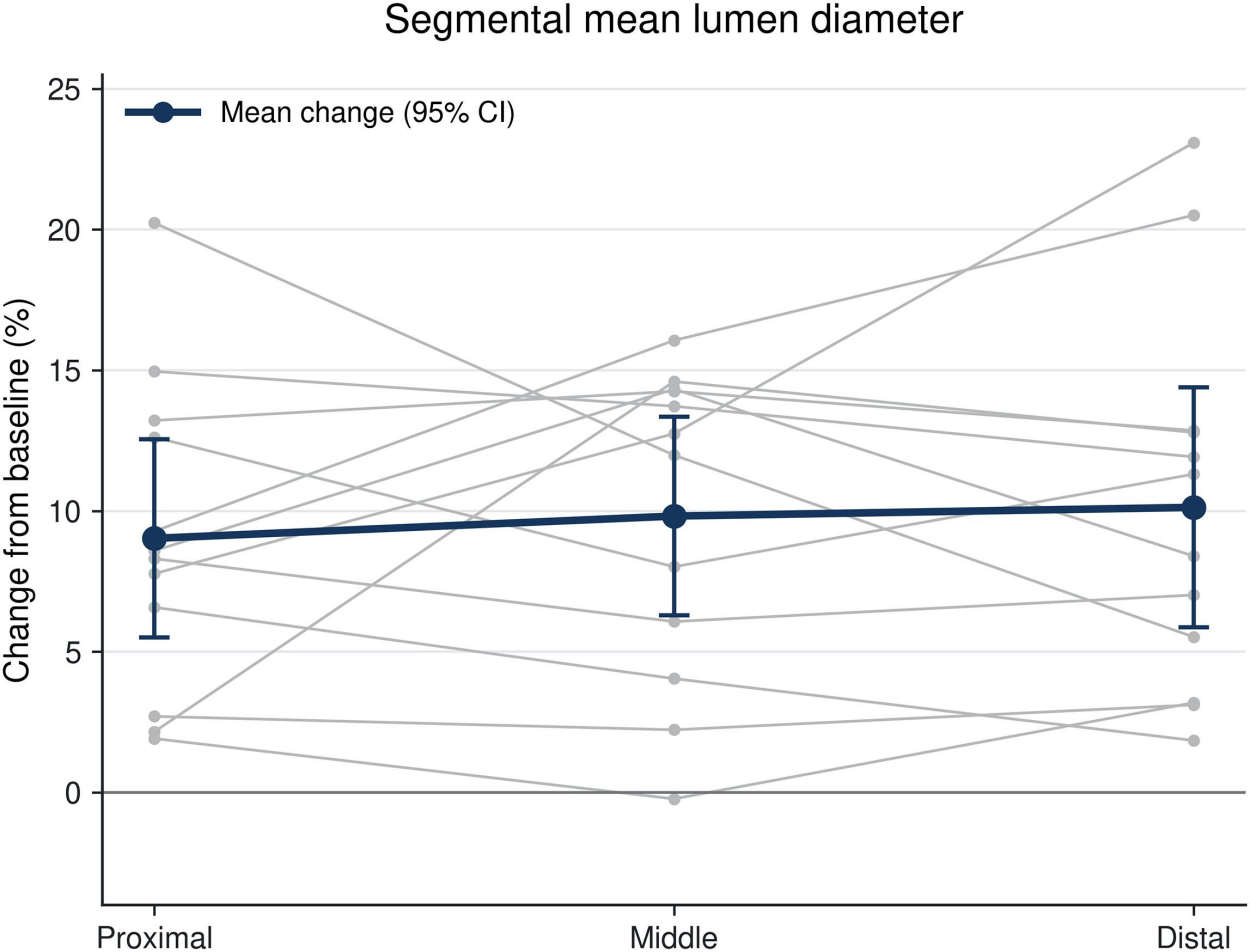
Segment-specific relative changes in mean radial artery lumen diameter after intra-arterial nitroglycerin and verapamil. Gray lines connect each patient's values across the proximal, middle, and distal thirds. Navy symbols and error bars show the mean relative change and its 95% confidence interval.

In an exploratory analysis, the narrowest distal minimum lumen diameter-to-sheath outer diameter ratio increased from 0.87 ± 0.19 at baseline to 1.04 ± 0.21 after administration (absolute change, 0.17 ± 0.13; relative increase, 20.6% ± 17.7%; *P* < 0.001). The ratio was <1 in 9 of 12 patients at baseline and 5 of 12 after administration; four patients crossed from <1 to ≥1.

## Discussion

The principal observation was a larger mean radial artery lumen diameter after intra-arterial nitroglycerin and verapamil. This within-patient difference should be interpreted alongside the study's timing, acquisition, and background-treatment limitations.

### Prior methods and evidence for radial vasodilation assessment

Methods used to assess radial artery vasodilation have varied across studies. Much of the available evidence comes from ultrasound, which is noninvasive and well suited for preprocedural diameter measurement and physiologic testing, including flow-mediated dilatation (FMD) and prolonged occlusion flow-mediated dilatation (PO-FMD) (3, 7, 11, 12). Angiography and quantitative angiographic analysis provide intraprocedural estimates of radial artery caliber under catheterization conditions and can assess diameter changes at spastic segments after intra-arterial vasodilator administration (1).

These approaches have been clinically useful, but they have important limitations. Ultrasound and angiography generally rely on selected measurement sites, are subject to operator dependence, and provide limited information on vessel wall structure along the radial artery (1, 3, 7, 11). Intravascular imaging offers complementary information. IVUS allows full-wall assessment with quantitative lumen and vessel measurements after transradial instrumentation (13), whereas OCT provides higher spatial resolution and has been used to characterize radial artery wall morphology and access-related vascular changes (8, 9). However, matched intravascular assessment of acute radial artery vasodilation across an extended analyzable vessel segment, with simultaneous quantification of lumen and vessel wall changes, remains limited (1, 3, 4, 7–13).

### Comparison with other imaging methods

OCT enabled matched cross-sectional assessment of mean lumen diameter across an extended radial artery span, with lumen area, volume, and vessel wall measurements providing supporting geometric information. The mean patient-level diameter difference was 0.29 mm (95% CI, 0.20–0.39), corresponding to a mean relative increase of 9.6%. These measurements describe the observed before-and-after difference and do not isolate the contribution of vasodilator administration.

These findings are broadly consistent with prior studies, although the magnitude of vasodilation differs across imaging modalities and study settings. In a preprocedural ultrasound study, sublingual nitroglycerin increased right radial artery diameter by 0.48 mm in men and 0.51 mm in women, corresponding to relative increases of approximately 16.7% and 22.9%, respectively (7). In a distal radial angiographic study performed after sheath insertion, nitroglycerin and verapamil increased the diameter of the most spastic segment by 0.35 mm and the reference diameter by 0.24 mm (1). In an IVUS volumetric study, intra-arterial nitroglycerin and verapamil increased radial artery diameter by 0.20 mm and lumen volume by 57 mm^3^, corresponding to relative increases of 6.6% and 14%, respectively (5).

The differences among studies should be interpreted in light of methodological and population-related factors. Ultrasound is typically performed before catheterization, angiography reflects intraprocedural vascular caliber but is projection-based, and IVUS and OCT differ in spatial resolution and lumen border definition. For lumen volume, direct comparison is particularly difficult because absolute volume depends on the analyzed vessel length, included arterial segments, baseline vessel size, and imaging method. The small sample size of the present study may also have contributed to variability in the estimated magnitude of vasodilation.

Unlike previous radial OCT studies focused on access-related injury or chronic structural change, the present study used matched before-and-after imaging across an extended analyzable segment. It adds to prior radial OCT studies focused on chronic structural changes after instrumentation or acute access-related injury (8, 9).

Standardized acquisition and measurement are central to quantitative OCT (14). Coronary repeated-pullback studies have reported reproducible lumen measurements (15, 16), but do not establish repeatability of the present multi-pullback radial artery workflow, which also requires catheter repositioning, saline flushing, stitching, and anatomical matching.

### Segmental radial artery findings and procedural implications

Mean lumen diameter was larger after administration in all three predefined thirds. The proximal third remained the largest and the distal third the smallest at both time points, preserving the observed proximal-to-distal caliber gradient.

This observation is consistent with anatomic data showing larger proximal than distal radial artery diameters along the vessel course (17). Ultrasound studies have also shown substantial interindividual variability in radial artery caliber, which is relevant to access planning (18). Differences in within-segment statistical significance do not demonstrate regional heterogeneity. The segmental findings are descriptive; sequential acquisition may also couple anatomical location with time after administration.

Systemic hemodynamic changes may influence arterial dimensions through changes in transmural pressure. Systolic arterial pressure decreased after administration, but the paired observational measurements cannot separate pressure-related effects from vasomotor, procedural, or time-related influences.

In our cohort, the oral calcium channel blockers were amlodipine and nifedipine, both dihydropyridines. Background nitrate and calcium channel blocker use may have influenced baseline vascular tone and the observed diameter change (19, 20). Although the paired design accounts for stable between-patient differences, it does not exclude modification of the acute response by background therapy.

### Clinical implications

The observed increase in radial artery diameter may be relevant to sheath-to-artery compatibility, particularly in the distal third of the imaged artery, which was closest to the puncture- and sheath-related zone. However, this small imaging study was not designed to assess procedural benefit or compare vasodilator regimens. The clinical relevance of these findings requires confirmation in larger studies with procedural endpoints.

### Limitations

This study has several limitations. The small, selected, relatively young and predominantly male cohort limited generalizability and statistical power. Baseline OCT obtained 5 min after puncture represented an early post-access rather than native preprocedural state, despite limiting sheath insertion to approximately 2 cm. Post-puncture spasm resolution, tissue warming, declining sympathetic tone, sedation, and saline flushing could have contributed to the observed change. The exploratory timing analysis cannot exclude these influences given the small sample, narrow interval range, absence of an untreated time-control series, and lack of a separately recorded drug-completion time.

No end-to-end test–retest protocol was performed. The observed 0.29-mm increase in mean lumen diameter therefore cannot be fully separated from acquisition, repositioning, flushing, stitching, and matching variability. Coronary repeatability data provide context (15, 16), but cannot establish that this change exceeds variability in the radial artery workflow.

Background vasoactive therapy may have influenced baseline tone and the acute diameter change. The small sample and incomplete last-dose timing precluded reliable medication-specific analyses. No segment-by-timepoint interaction was tested. Without pullback-level timestamps, anatomical differences cannot be separated from time-dependent changes.

Nitroglycerin and verapamil were administered together, precluding assessment of their individual effects. A single post-administration series captured only an early observation period and could not establish the peak response or complete time course. Repeated catheter passage could also have influenced vascular tone. Without continuous pressure monitoring, the effects of intervening hemodynamic changes could not be quantified. Clinical implications for procedural outcomes, long-term radial artery remodeling, and vessel patency remain uncertain. Additional cost and procedural burden may limit routine radial artery OCT use.

## Conclusions

Mean radial artery lumen diameter was greater after intra-arterial nitroglycerin and verapamil than at baseline.

## Data Availability

The raw data supporting the conclusions of this article will be made available by the authors, without undue reservation.
