# Supplementary material for: Radial artery characteristics before and after intra-arterial administration of nitroglycerin and verapamil: an optical coherence tomography study
Source: Front Med (Lausanne). 2026 Sep 29;13:1896387. doi: 10.3389/fmed.2026.1896387 (PMC13658102; doi:10.3389/fmed.2026.1896387)
Supplement: Supplementary file 1 [file Table1.docx]

**Supplementary Table 1.** Overall interobserver reproducibility of OCT-derived radial artery lumen and wall measurements

| **Parameter** | **ICC(2,1)** | **95% CI** |
| --- | --- | --- |
| Lumen area | 0.999 | 0.999–0.999 |
| Mean lumen diameter | 0.999 | 0.999–1.000 |
| Minimum lumen diameter | 0.999 | 0.998–0.999 |
| Maximum lumen diameter | 0.999 | 0.998–0.999 |
| IEL area | 0.999 | 0.998–0.999 |
| EEL area | 0.998 | 0.997–0.999 |
| Medial area | 0.949 | 0.918–0.968 |

Values are ICCs with 95% CIs. Interobserver reproducibility was assessed in 12 patients using 10 randomly selected OCT cross-sectional frames per patient (120 frames in total). ICCs were calculated using a two-way random-effects, absolute-agreement, single-measure model [ICC(2,1)]. CI, confidence interval; EEL, external elastic lamina; ICC, intraclass correlation coefficient; IEL, internal elastic lamina; OCT, optical coherence tomography.

**Supplementary Table 2.** Interobserver reproducibility of OCT-derived measurements by radial artery segment

| **Segment** | **OCT-derived measurement** | **Baseline ICC (95% CI)** | **Post-vasodilator ICC (95% CI)** |
| --- | --- | --- | --- |
| **Proximal** | Lumen area, mm² | 1.000 (0.999–1.000) | 0.999 (0.997–1.000) |
|  | Mean lumen diameter, mm | 1.000 (0.999–1.000) | 0.999 (0.997–1.000) |
|  | Minimum lumen diameter, mm | 1.000 (0.998–1.000) | 0.998 (0.993–0.999) |
|  | Maximum lumen diameter, mm | 0.999 (0.996–1.000) | 0.998 (0.994–0.999) |
|  | IEL area, mm² | 0.999 (0.997–1.000) | 0.999 (0.995–1.000) |
|  | EEL area, mm² | 0.999 (0.994–1.000) | 0.998 (0.983–0.999) |
|  | Medial area, mm² | 0.956 (0.860–0.987) | 0.976 (0.893–0.993) |
| **Middle** | Lumen area, mm² | 0.999 (0.997–1.000) | 0.999 (0.996–1.000) |
|  | Mean lumen diameter, mm | 0.999 (0.998–1.000) | 0.999 (0.995–1.000) |
|  | Minimum lumen diameter, mm | 0.998 (0.994–1.000) | 0.999 (0.996–1.000) |
|  | Maximum lumen diameter, mm | 0.999 (0.996–1.000) | 0.998 (0.995–1.000) |
|  | IEL area, mm² | 0.998 (0.995–1.000) | 0.998 (0.993–0.999) |
|  | EEL area, mm² | 0.998 (0.994–1.000) | 0.998 (0.991–0.999) |
|  | Medial area, mm² | 0.975 (0.918–0.993) | 0.961 (0.869–0.989) |
| **Distal** | Lumen area, mm² | 0.999 (0.998–1.000) | 0.998 (0.994–1.000) |
|  | Mean lumen diameter, mm | 1.000 (0.998–1.000) | 0.999 (0.994–1.000) |
|  | Minimum lumen diameter, mm | 0.999 (0.997–1.000) | 0.998 (0.994–0.999) |
|  | Maximum lumen diameter, mm | 1.000 (0.999–1.000) | 0.999 (0.994–1.000) |
|  | IEL area, mm² | 0.999 (0.997–1.000) | 0.999 (0.995–1.000) |
|  | EEL area, mm² | 0.999 (0.995–1.000) | 0.998 (0.994–0.999) |
|  | Medial area, mm² | 0.977 (0.923–0.993) | 0.984 (0.941–0.996) |

Values are ICCs (95% CIs). ICCs were calculated using a two-way random-effects, absolute-agreement, single-measure model [ICC(2,1)] based on measurements from 12 patients independently assessed by two observers. CI, confidence interval; EEL, external elastic lamina; ICC, intraclass correlation coefficient; IEL, internal elastic lamina; OCT, optical coherence tomography.

**Supplementary Table 3.** Interobserver reproducibility of longitudinal OCT anatomic matching

| **Parameter** | **Observer 1** | **Observer 2** | **Mean absolute difference, frames** | **ICC (95% CI)** |
| --- | --- | --- | --- | --- |
| Proximal matching point (start frame) | 28.9 ± 25.0 | 28.9 ± 25.0 | 0.00 | 1.000 (1.000–1.000) |
| Distal matching point (end frame) | 226.5 ± 27.2 | 226.8 ± 27.4 | 0.46 | 0.999 (0.998–1.000) |
| Matched OCT segment length, frames | 197.7 ± 34.0 | 197.9 ± 34.3 | 0.46 | 0.999 (0.999–1.000) |

Observer values are presented as mean ± SD; mean absolute differences are expressed in frames; and ICCs are reported with 95% CIs. Reproducibility was assessed in all 12 patients using 24 condition-specific reconstructed OCT datasets, comprising baseline and post-vasodilator datasets for each patient. Each dataset was reconstructed from three or four sequential pullbacks. ICCs were calculated using a two-way random-effects, absolute-agreement, single-measure model [ICC(2,1)]. CI, confidence interval; ICC, intraclass correlation coefficient; OCT, optical coherence tomography; SD, standard deviation.
